# Age-disparate relationships and HIV incidence in adolescent girls and young women: evidence from Zimbabwe

**DOI:** 10.1097/QAD.0000000000001506

**Published:** 2017-06-02

**Authors:** Robin Schaefer, Simon Gregson, Jeffrey W. Eaton, Owen Mugurungi, Rebecca Rhead, Albert Takaruza, Rufurwokuda Maswera, Constance Nyamukapa

**Affiliations:** a Department of Infectious Disease Epidemiology, Imperial College London, Norfolk Place, London, UK; b Biomedical Research and Training Institute; c Zimbabwe Ministry of Health and Child Care, Harare, Zimbabwe.

**Keywords:** adolescent girls, age-disparate relationships, HIV incidence, HIV prevention, older men, young women

## Abstract

**Objective:**

Age-disparate sexual relationships with older men may drive high rates of HIV acquisition in young women in sub-Saharan Africa, but evidence is limited. We investigate the association between age-disparate relationships and HIV incidence in Manicaland, Zimbabwe.

**Design:**

A general-population open-cohort study (six surveys) (1998–2013).

**Methods:**

A total of 3746 young women aged 15–24 years participated in consecutive surveys and were HIV-negative at the beginning of intersurvey periods. Last sexual partner age difference and age-disparate relationships [intergenerational (≥10 years age difference) and intragenerational (5–9 years) versus age-homogeneous (0–4 years)] were tested for associations with HIV incidence in Cox regressions. A proximate determinants framework was used to explore factors possibly explaining variations in the contribution of age-disparate relationships to HIV incidence between populations and over time.

**Results:**

About 126 HIV infections occurred over 8777 person-years (1.43 per 100 person-years; 95% confidence interval = 1.17–1.68). Sixty-five percent of women reported partner age differences of at least 5 years. Increasing partner age differences were associated with higher HIV incidence [adjusted hazard ratio (aHR) = 1.05 (1.01–1.09)]. Intergenerational relationships tended to increase HIV incidence [aHR = 1.78 (0.96–3.29)] but not intragenerational relationships [aHR = 0.91 (0.47–1.76)]. Secondary education was associated with reductions in intergenerational relationships [adjusted odds ratio (aOR) = 0.49 (0.36–0.68)]. Intergenerational relationships were associated with partners having concurrent relationships [aOR = 2.59 (1.81–3.70)], which tended to increase HIV incidence [aHR = 1.74 (0.96–3.17)]. Associations between age disparity and HIV incidence did not change over time.

**Conclusion:**

Sexual relationships with older men expose young women to increased risk of HIV acquisition in Manicaland, which did not change over time, even with introduction of antiretroviral therapy.

## Abstract 2

Supplemental Digital Content is available in the text

## Introduction

HIV infection rates remain extremely high in adolescent girls and young women in many populations in sub-Saharan Africa. In South Africa and Zimbabwe, for example, HIV incidence in women aged 15–24 years is four times higher and twice as high, respectively, as in men of the same age [1,2]. These high infection rates present huge challenges for the health and wellbeing of these women and for the affordability and sustainability of national antiretroviral treatment (ART) programmes. More effective prevention interventions are therefore needed urgently and must be founded on a better understanding of the complex social and biological drivers of HIV infection in young women [3].

Mathematical modelling studies show that sexual relationships with older men (age-disparate relationships) can be a major driver of HIV infection in young women [4]. As HIV prevalence is higher among older men than in younger men [3], young women engaging in sexual relations with older men can be at a greater risk of contracting HIV compared with those forming relationships with similarly aged men. Moreover, age-disparate sexual relationships are often characterized by socio-economic asymmetries, which can leave young women unable to negotiate condom use or vulnerable to forced sex [5,6].

Cross-sectional studies from several countries have provided empirical evidence that age-disparate sexual relationships are common in African populations and that HIV prevalence is higher among young women who report these relationships [refer to Supplemental Digital Content (SDC) 1, http://links.lww.com/QAD/B92 for a review of previous studies] [7–12]. However, data on HIV incidence from longitudinal cohort studies are needed to provide more conclusive evidence that age-disparate relationships contribute to the high rates of HIV acquisition found in young women. Unfortunately, to date, only a small number of cohort studies have been conducted, and the evidence from these studies is mixed. A study in Uganda found a positive association between having a partner more than 15 years older and HIV incidence, but this study was restricted to married women and was conducted largely before ART was widely available [13]. No association was found in a large general-population study in KwaZulu-Natal, South Africa [14], or in secondary analyses of trial data in Uganda and South Africa [8,15,16].

The mixed results from these longitudinal studies may be due to differences or limitations in study design such as inclusion of older women, short follow-up periods, and trial settings. However, they could also reflect genuine variation in the effects of age-disparate relationships on HIV incidence between populations or over time. Reasons for such variation could include differences in the extent of age-disparate relationships between populations, differences in the age pattern of HIV prevalence in men, and differences in the prevalence of factors that lie on causal pathways between age-disparate relationships and HIV incidence. The latter could include factors affecting levels of HIV viral load and infectiousness in partners with HIV (e.g. concurrent partnerships or ART uptake and adherence) as well as use of condoms and other HIV prevention methods. However, the extent of these factors within age-disparate relationships and their influence on the risk of HIV acquisition is currently not well understood.

The objectives of this study are to use data from a long-running general-population cohort in eastern Zimbabwe, which spans the introduction and scale-up of ART services, (1) to establish whether age-disparate relationships are associated with increased HIV incidence in young women in a high HIV prevalence setting in sub-Saharan Africa, and (2) to investigate factors that may explain variations in the contribution of age-disparate relationships to the spread of HIV infection among young women between populations and over time. For the latter, we use an adaptation of the proximate determinants framework to investigate underlying factors associated with levels of occurrence of age-disparate relationships and proximate factors that may mediate between age-disparate relationships and HIV incidence (refer to SDC 2, http://links.lww.com/QAD/B92 for details) [17].

## Methods

### Data source

Between 1998 and 2013, the Manicaland HIV/STD Prevention Project (Manicaland Project) completed six rounds of a general-population open-cohort survey, including HIV sero-testing, in three districts in Manicaland, Zimbabwe, covering demographic and socio-economic characteristics, sexual behaviour, HIV/AIDS-related knowledge, and uptake of HIV services (http://www.manicalandhivproject.org/ and Gregson *et al*. [18]). Participants for individual interviews were selected from a household census in 12 sites (eight in survey round 6), representing small towns, farming estates, roadside trading centres, and subsistence farming villages. Surveys included between 8000 and 15 000 adults aged 15–54 years, and participation rates ranged between 73.0 and 79.5%. Nonparticipation was mainly due to temporary absences from the households, although up to three attempts were made to reach eligible persons. Intervals between surveys were about 3 years, and follow-up rates ranged between 47.0 and 60.6%. Most loss-to-follow-up was due to participants becoming ineligible due to out-migration or death. Among those who remained eligible, follow-up ranged from 77.0 to 96.4%. Ethical approval for the Manicaland Project was obtained from the Imperial College London Research Ethics Committee and the Medical Research Council of Zimbabwe.

### Data analysis

For inclusion in this study, participants in the Manicaland cohort had to fulfil the following criteria at the beginning of an intersurvey period: (1) female, (2), aged 15–24 years, (3), participated in at least two consecutive survey rounds, and (4) HIV-negative at the beginning of an interval between surveys (refer to SDC 3, http://links.lww.com/QAD/B92 for a discussion of the inclusion criteria). Young women who participated in more than two surveys were included with each intersurvey period being treated as a separate observation, so there are more observations than participants. This counting process of separate observations with no overlapping time periods allowed for time-varying covariates and ensured that there was no correlation of observations from the same individual [19,20]. Those who participated in several surveys but were not seen in one or more surveys in between were not included given the long intervals between interviews. For each variable tested for association with HIV incidence, data were used from the survey at the beginning of an interval between rounds. Participants were right-censored at their last survey participation date or 25th birthday. Analyses including sexual behaviour variables were restricted to participants who reported having started sex at the beginning of the intersurvey period; all other analyses included all women.

The HIV infection date for those who became HIV-positive between surveys was unknown. As using the mid-point date between surveys can introduce bias [21,22], a multiple imputation approach was used [21]. In this, a different random HIV infection date between surveys was imputed for each of 30 datasets. An imputed seroconversion date may be after the age-censoring date, so the number of HIV infections varies slightly between datasets. Analyses were conducted for each imputed dataset, and results were pooled according to Rubin's rules [23]. For confidence intervals (CIs) and significance tests, a Student's *t* approximation was used [23].

Based on the self-reported age of the most recent sexual partner, age difference to the partner (in years) was investigated as a continuous variable. Sexual partnerships were also divided into age-homogenous (0–4 years age difference), intragenerational (5–9 years), and intergenerational relationships (≥10 years) [5,8]. Young women reporting younger partners (*n* = 21) were included in these categories (20 had 0–4 years younger partners; one partner was 6 years younger). In survey rounds 1 and 2, only data of the last partner in the past month were collected, whereas this time restriction was removed from round 3. Associations with incident HIV infection were tested using Cox proportional hazard's regression including survey round as a covariate to control for unmeasured temporal confounders and changes in infection risks over time. To test for a crude association between age-disparate relationships and HIV incidence, a Cox model including age-disparate relationships and age was estimated. Preliminary analysis, in which various background characteristics were tested for associations with HIV incidence (SDC 4, http://links.lww.com/QAD/B92), indicated marital status as a possible additional confounding factor, so a further model was developed to include marital status. Models were estimated with cluster-robust standard error estimation, using standardized village names as the clustering unit. Subanalyses were conducted to establish whether the association varied by socio-demographic characteristics by using interaction terms with these in Cox models. Participants missing data on the age of the last partner and those lost to follow-up were examined (SDC 5, http://links.lww.com/QAD/B92). Data on marital partners and the age of the last sexual partner at the end of the period between surveys were also analysed (SDC 5, http://links.lww.com/QAD/B92).

**Fig. 1:**
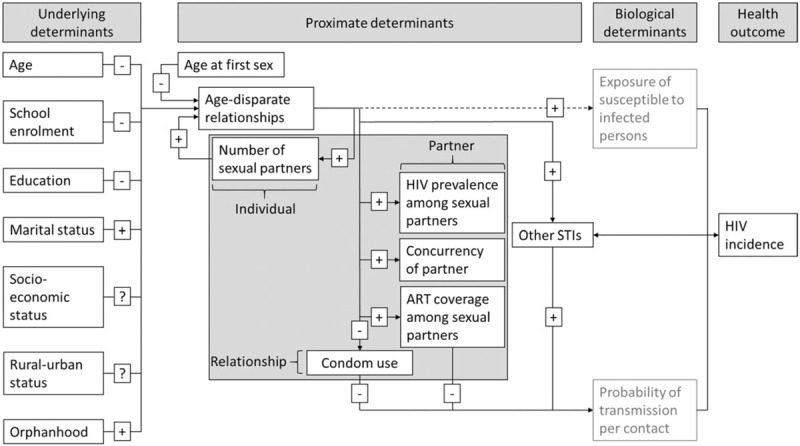
The proximate determinants framework for HIV infection with focus on age-disparate relationships.

For analyses of factors that may contribute to variation in the association between age-disparate relationships and HIV incidence in young women over time and between populations, an adaptation of the proximate determinants framework for HIV acquisition was used (Fig. 1) [17,24]. In this version of the framework, the occurrence of age-disparate relationships is hypothesized to be determined by underlying socio-demographic factors including age, education, and socio-economic status. Then, age-disparate relationships are conceptualized as mediators between these underlying determinants and HIV infection, and other proximate determinants are conceptualized to mediate between age-disparate relationships and HIV infection. Refer to SDC 2, http://links.lww.com/QAD/B92 for details on the framework and the underlying data.

Age-disparate relationships are conceptualized as mediators between the underlying determinants and HIV infection, and other proximate determinants (in the grey box) are conceptualized to mediate between age-disparate relationships and HIV infection. These proximate determinants can be distinguished between those relating to the individual, those relating to the partner, and those relating to the relationship itself. The biological determinants are not measured directly. Other sexually transmitted infections are separate from other proximate determinants as they are themselves an outcome of the other proximate determinants as well as a determinant of HIV infection. The signs in the boxes on the hypothesized causal pathways indicate the expected direction of association with age-disparate relationships or with biological determinants based on the published literature. This is discussed in detail, including references, in SDC 2, http://links.lww.com/QAD/B92. The pathway between age-disparate relationships and exposure to infected persons is indirect (dashed line) through other proximate determinants in the grey box. Other pathways linking the underlying determinants to HIV infection are not indicated.

To establish whether levels of age-disparate relationships vary by socio-demographic characteristics in the current study population, occurrence of age-disparate relationships was compared between women with different characteristics using logistic regression. Age-disparate relationships as the outcome measure for this were disaggregated into ‘5–9 years age difference’ and ‘≥10 years’, respectively, against the reference category ‘0–4 years difference’.

To explore the potential importance of factors that could lie on causal pathways between age-disparate relationships and HIV incidence in contributing to variation between populations in the strength of this association, age-disparate relationships were tested for association with each proximate determinant using logistic regression with the same outcome measures as for the socio-demographic characteristics, and each determinant was tested for association with HIV incidence using Cox regression. Where a proximate determinant was found to be associated with both age-disparate relationships and HIV incidence, a Cox model was estimated to test whether inclusion of this factor in the regression weakened the association between age-disparate relationships and HIV incidence (i.e. indicating that the factor may lie on a causal pathway).

No data were available on partner HIV infection and ART uptake status for young women in the study; therefore, the mediating role of ART could not be evaluated directly. ART scale-up began in Zimbabwe in 2005 and was escalated after 2009. To test the hypothesis that the association between age disparity and HIV incidence weakens with increasing ART availability, time period was used as a proxy for ART coverage and viral load suppression among partners. A time period variable was created to include all participants with intersurvey periods starting before 2005 (pre-ART) and from 2005 (post-ART). Another variable was created dividing time into before 2005 (pre-ART), 2005–2008 (ART scale-up), and after 2009 (post-ART). The interaction between these variables and age-disparate relationships was tested in Cox models adjusted for age and marital status.

## Results

### Trends in HIV infection rates and age-disparate relationships

**Fig. 2:**
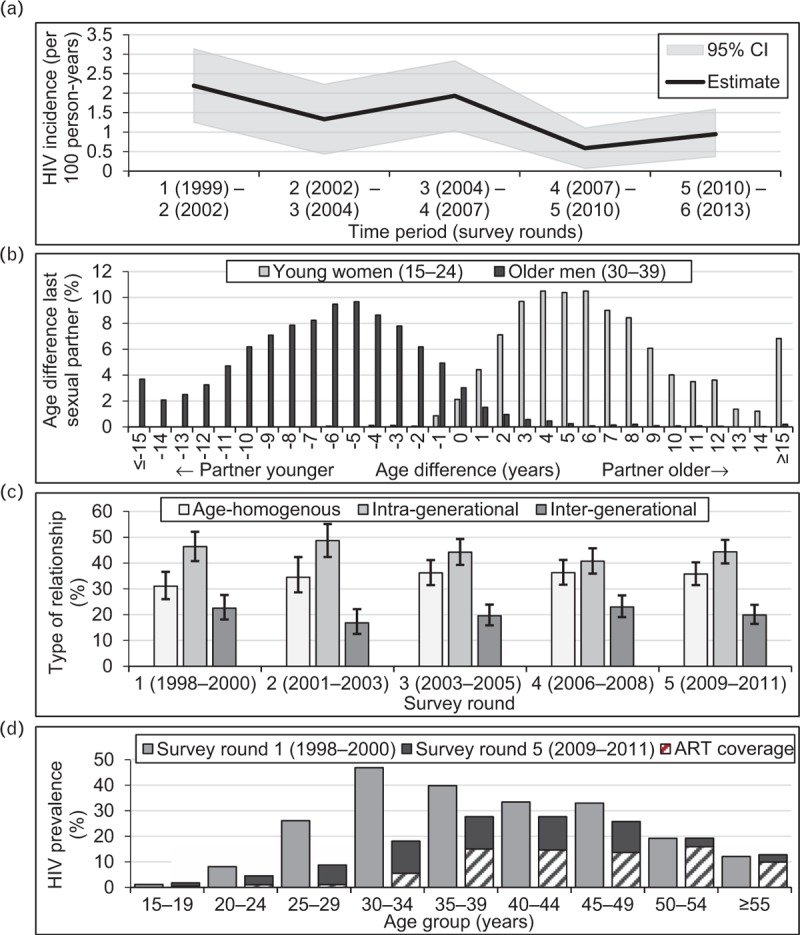
HIV incidence in young women (15–24 years), age-disparate relationships in young women and older men (30–39 years), and age-specific HIV prevalence in men; Manicaland, Zimbabwe, 1998–2013.

A total of 3082 young women aged 15–24 years met the inclusion criteria for the study, contributing 3746 observations. Tables 2 and 3 include descriptive statistics for this sample. A total of 126 new HIV infections occurred over 8777 person-years (1.43 per 100 person-years, 95% CI = 1.17–1.68). HIV incidence rates followed a declining trend between 1998 and 2013 with a temporary increase in the mid-2000s, and rates remained high in the most recent intersurvey period [0.95 (0.37–1.59)] (Fig. 2a). A total of 2262 observations include young women reporting ever having had sex, and data on the most recent sexual partner were provided by 1734 of these, showing that 44.5% (42.1–46.8%) had partner age differences of 5–9 years and 20.5% (18.7–22.5%) had partners at least 10 years older (Fig. 2b). These proportions remained reasonably constant over time (Fig. 2c). HIV prevalence in men declined over time, with the distribution becoming increasingly skewed towards older ages; however, prevalence among men aged 30 years and above was consistently higher than prevalence among younger men (Fig. 2d). ART coverage was about 31% among HIV-positive men aged 30–34 years and over 50% among older male age groups in the survey round preceding the last follow-up (2009–2011).

(a) Age-standardized HIV incidence with 95% confidence interval (CI) over survey rounds among young women (15–24 years) based on 30 imputed random dates of HIV infection between survey rounds. The years refer to mid-points of survey rounds. The data were directly age-standardized using the 2012 Manicaland census age structure. (b) Age differences to the last sexual partner among young women (15–24 years) and older men (30–39 years). Data for older men were pooled across all survey rounds and include HIV-positive and HIV-negative men. (c) Intergenerational (≥10 years age difference), intragenerational (5–9 years), and age-homogenous (0–4 years) relationships among young women (15–24 years) by survey round, based on age of last sexual partner. (d) HIV prevalence by 5-year age group in men in survey rounds 1 (1998–2000) and 5 (2009–2011). For survey round 5, diagonal lines indicate the proportion of HIV-positive men who have ever taken antiretroviral drugs (antiretroviral treatment coverage).

### Association between age-disparate relationships and HIV incidence

Increasing age difference to partner was associated with a higher HIV incidence in 15–24-year-old women [adjusted hazard ratio (aHR) = 1.05 per year older (1.01–1.09)] (adjusting for age, marital status, and survey round) (Table 1). HIV incidence tended to be higher in intergenerational relationships (≥10 years age difference) than in age-homogeneous relationships [2.56 per 100 person-years (1.37–3.75) versus 1.49 (0.78–2.20); aHR = 1.79 (0.98–3.28)] but there was no difference for intragenerational relationships [1.34 (0.76–1.93) versus 1.49 (0.78–2.20); aHR = 0.88 (0.45–1.73)] (adjusting for age, marital status, and survey round) (Table 1). This pattern of association between intergenerational relationships and HIV incidence held true when women who reported not having had sex in at least the past 12 months (who may have been at reduced risk of HIV infection) were excluded from the analysis [aHR = 1.96 (1.05–3.64)] (SDC 5, http://links.lww.com/QAD/B92) and in an analysis of women aged 15–29 years [aHR = 1.77 (1.17–2.68)] (SDC 6, http://links.lww.com/QAD/B92). The results were also similar when using the age of the marital partner rather than the age of the last sexual partner [aHR = 1.88 (0.774–4.576)] (SDC 5, http://links.lww.com/QAD/B92), suggesting that the reported last sexual partner is likely to be the marital partner.

**Table 1:** The association between age-disparate relationships and HIV incidence in young women (15–24 years), Manicaland, Zimbabwe, 1998–2013.

Variable		aHR (95% CI)	*P* value	aHR (95% CI)	*P* value
	Infections/pyrs (IR per 100 pyrs)	Model 1 (*n* = 1733)		Model 2 (*n* = 1718)	
Age difference to last partner		1.05 (1.01–1.09)	0.009	1.05 (1.01–1.09)	0.010
Age-disparate relationship (age difference in years)					
Age-homogenous (0–4)	18.6/1249 (1.49)	1 (baseline)		1 (baseline)	
Intragenerational (5–9)	22.1/1644 (1.34)	0.91 (0.47–1.76)	0.77	0.88 (0.45–1.73)	0.72
Intergenerational (≥10)	19.7/770 (2.56)	1.78 (0.96–3.29)	0.066	1.79 (0.98–3.28)	0.060

Values are new HIV infections per person-years (pyrs), incidence rates per 100 person-years (IR per 100 pyrs), adjusted hazard ratios (aHRs), 95% confidence intervals (CIs), and *P* values. Incidence rates are crude. Age difference to partner and age-disparate relationships were included in separate models but with the same covariates. Models 1 and 2 include age and survey round as covariates. Model 2 also includes marital status as a potential confounding variable. The covariate results are not shown. Results are based on 30 imputed random dates of HIV infection between surveys. Participants were censored at their 25th birthday.

Similar patterns of association between age-disparate relationships and HIV incidence were found irrespective of age group (15–19 and 20–24 years), socio-economic location (urban, periurban, and rural), household wealth, marital status, and orphanhood (SDC 4, http://links.lww.com/QAD/B92). When restricting the sample to married young women and using the same classification as the Ugandan cohort study of married women [13], women with partners more than 15 years older had higher HIV incidence than those with partners 0–15 years older [aHR = 3.55 (1.65–7.62)].

### Underlying determinants associated with the occurrence of age-disparate relationships

Table 2 shows the occurrence of age-disparate relationships among young women by underlying socio-demographic characteristics. Age-disparate relationships become less common as women become older, are less common in young women with greater school education, and are more common in currently married and divorced women than in never-married women. Those in the least poor household wealth quintile are more likely to be in age-disparate relationships, but the number of women in this group is small, and confidence intervals are wide. No differences in the occurrence of age-disparate relationships were found for urban–rural residence or between young women with and without surviving parents.

**Table 2:** Associations between age-disparate relationships and underlying determinants of HIV infection in young women (15–24 years), Manicaland, Zimbabwe, 1998–2013.

		Association with intragenerational relationships (5–9 years age difference)		Association with intergenerational relationships (≥10 years difference)	
Variable	*N* (%)	aOR (95% CI)	*P* value	aOR (95% CI)	*P* value
Age (years)		(*N* = 1378)		(*N* = 963)	
15–19	1941 (51.8)	1 (baseline)		1 (baseline)	
20–24	1805 (48.2)	0.52 (0.42–0.65)	<0.0001	0.55 (0.41–0.73)	<0.0001
School enrolment		(*N* = 1373)		(*N* = 960)	
Not enrolled	2717 (72.7)	1 (baseline)		1 (baseline)	
Enrolled	1020 (27.3)	0.52 (0.18–1.54)	0.24	0.62 (0.15–2.48)	0.50
Education		(*N* = 1375)		(*N* = 960)	
None/primary	755 (20.2)	1 (baseline)		1 (baseline)	
Secondary/higher	2983 (79.8)	0.71 (0.53–0.94)	0.019	0.49 (0.36–0.68)	<0.0001
Marital status		(*N* = 1365)		(*N* = 957)	
Never married	1506 (42.2)	1 (baseline)		1 (baseline)	
Currently married	1842 (51.6)	2.45 (1.44–4.18)	0.001	3.21 (1.51–6.82)	0.002
Divorced/separated	199 (5.6)	2.25 (1.12–4.53)	0.023	2.86 (1.10–7.45)	0.032
Widowed	23 (0.6)	0.37 (0.04–3.53)	0.385	2.82 (0.66–12.1)	0.16
Household wealth index (quintiles)		(*N* = 1359)		(*N* = 948)	
Poorest	510 (13.8)	1 (baseline)		1 (baseline)	
2nd poorest	1805 (48.8)	0.95 (0.69–1.31)	0.76	1.10 (0.76–1.60)	0.62
Middle	1062 (28.7)	0.98 (0.69–1.37)	0.89	1.06 (0.70–1.61)	0.77
2nd highest	285 (7.7)	0.69 (0.41–1.16)	0.16	0.62 (0.33–1.17)	0.14
Least poor	39 (1.1)	2.39 (0.65–8.73)	0.19	3.77 (1.09–13.0)	0.036
Rural–urban residence status (distance to town)		(*N* = 1308)		(*N* = 906)	
Urban (0–4 km)	770 (21.5)	1 (baseline)		1 (baseline)	
Periurban (5–9 km)	561 (15.6)	1.04 (0.73–1.49)	0.83	1.10 (0.67–1.78)	0.71
Rural (≥10 km)	2259 (62.9)	0.94 (0.73–1.20)	0.61	0.93 (0.66–1.32)	0.70
Orphan type^a^		(*N* = 1027)		(*N* = 717)	
No orphan	1586 (56.6)	1 (baseline)		1 (baseline)	
Paternal	665 (23.7)	1.09 (0.80–1.48)	0.57	0.97 (0.63–1.50)	0.89
Maternal	213 (7.6)	1.16 (0.77–1.85)	0.43	1.05 (0.59–1.85)	0.87
Double	337 (12.0)	1.24 (0.83–1.86)	0.29	1.39 (0.83–2.34)	0.21

The column on sample sizes (*N*) refers to the sample of young women as a whole; sample sizes in brackets in the columns on the associations with age-disparate relationships refer to the sample sizes for each logistic regression model. Numbers may not add up to 100% due to rounding. The values for the tests for association (logistic regressions) are adjusted odds ratios (aORs), 95% confidence intervals (CIs), and *P* values. The reference category for the outcome variable was ‘age-homogenous relationship (0–4 years age difference)’. Each variable was tested in a separate model including age and survey round as covariates.

^a^Data on orphan type were not available for survey round 1. In round 2, orphanhood data were only available for those aged under 19 years. From round 3, the data were available for all participants. For the analyses, the data were pooled despite the inconsistencies in measurement.

### Proximate determinants mediating between age-disparate relationships and HIV incidence

Other proximate determinants of HIV infection may contribute to the association between age-disparate relationships and HIV incidence if they lie on causal pathways that contribute to this association (Fig. 1). Table 3 shows the results on patterns of association between intergenerational relationships and the proximate determinants measured in the study, and associations between these proximate determinants and HIV incidence. Evidence was found for associations between intergenerational relationships and concurrency of partner (partner is married to another woman) [age-adjusted odds ratio = 2.59 (1.81–3.70)]. There was also some evidence for a possible association between concurrency of partner and HIV incidence [aHR = 1.74 (0.96–3.17)]. The association between intergenerational relationships and HIV incidence differed strongly between those who reported that their partners had other partners [aHR = 4.64 (0.72–29.7)] and those who did not report this [aHR = 1.35 (0.67–2.74)]. In a Cox regression adjusting for concurrency of partner and woman's age, the effect size and significance of the association between intergenerational relationships and HIV incidence were reduced [aHR = 1.65 (0.91–3.01)], suggesting that concurrency of partner may contribute to this association.

**Table 3:** Associations between age-disparate relationships, proximate determinants of HIV infection, and HIV incidence in young women (15–24 years), Manicaland, Zimbabwe, 1998–2013.

		Association with intergenerational relationships (≥10 years difference)		Association with HIV incidence	
Variable	*N* (%)	aOR (95% CI)	*P* value	aHR (95% CI)	*P* value
STI symptoms in past 12 months		(*N* = 958)		(*N* = 2257)	
No symptoms	1936 (85.8)	1 (baseline)		1 (baseline)	
STI symptoms	321 (14.2)	1.14 (0.76–1.72)	0.52	1.31 (0.76–2.28)	0.33
Multiple partners in past 12 months		(*N* = 959)		(*N* = 2252)	
0–1 partner	2189 (97.2)	1 (baseline)		1 (baseline)	
>1 partner	63 (2.8)	1.03 (0.45–2.35)	0.95	1.82 (0.75–4.43)	0.19
Concurrency of partner		(*N* = 960)		(*N* = 1770)	
No other wives	1481 (83.7)	1 (baseline)		1 (baseline)	
Other wives	289 (16.3)	2.59 (1.81–3.70)	<0.0001	1.74 (0.96–3.17)	0.069
Condom use (past 2 weeks)^a^		(*N* = 948)		(*N* = 1753)	
No consistent use	1295 (73.9)	1 (baseline)		1 (baseline)	
Consistent use	77 (4.4)	0.97 (0.52–1.80)	0.92	0.60 (0.09–4.19)	0.61
NA	381 (21.7)	0.72 (0.51–1.01)	0.057	1.74 (0.98–3.11)	0.059
Condom use (last sex)^b^		(*N* = 685)		(*N* = 1435)	
No condom used	1296 (90.3)	1 (baseline)		1 (baseline)	
Condom used	139 (9.7)	0.84 (0.50–1.43)	0.53	1.39 (0.56–3.46)	0.48

The column on sample sizes (*N*) refers to the sample of young women as a whole; sample sizes in brackets in the columns on the associations with age-disparate relationships and HIV incidence refer to the sample sizes for each logistic and Cox regression model, respectively. Numbers may not add up to 100% due to rounding. The values for the tests for association are adjusted odds ratios (aORs) (for logistic regressions) and adjusted hazard ratios (aHRs) (for Cox regressions), 95% confidence intervals (CIs), and *P* values. The reference category for the outcome variable in logistic regressions was ‘age-homogenous relationship (0–4 years age difference)’. Cox regression results are based on 30 imputed random dates of HIV infection between survey rounds and participants were censored at the 25th birthday. Each variable was tested in separate models including age and survey round as covariates.

^a^This condom use variable was created on the basis of the number of times a person reported to have had sex in the past 2 weeks and the number of times a condom was used in the past 2 weeks. Consistent condom use was defined as having used a condom during each time the respondent has had sex in the past 2 weeks. Persons reporting not having had sex in the past 2 weeks are classified as ‘NA’ (not applicable).

^b^This condom use variable was based on the direct question of whether a condom was used during the last time the participant had sex, which was asked from survey round 3 onwards.

There was no change in the age-adjusted hazard of HIV acquisition in intergenerational relationships over time [pre-ART (1998–2004) aHR = 1.89 (0.89–3.99); post-ART (2005–2011) aHR = 1.73 (0.61–4.94)], and the interaction between age-disparate relationships and time period was weak (*P* > 0.5; refer to SDC 7, http://links.lww.com/QAD/B92 for details).

## Discussion

In a large cohort study in east Zimbabwe, we found that age-disparate sexual relationships are associated with increased HIV incidence among young women aged 15–24 years, particularly where partners are 10 or more years older. Despite overall declines in HIV prevalence over time, older men had consistently a higher HIV prevalence than younger men, thus exposing young women to an increased risk of HIV infection, particularly given the generally low levels of condom use in the study population. Although only a minority of young women reported partners 10 or more years older, the proportion is still substantial (21%) and similar to Zimbabwe as a whole [25], so these intergenerational relationships are still likely to be an important contributor to new infections in young women and to the HIV epidemic in this setting, especially when considering the future onward transmission from young women (including possibly to their children). The increased risk was found in all age groups (including 25–29-year-olds).

As far as we are aware, this is the first study to demonstrate an association between increasing partner age and HIV incidence in a representative general-population sample covering pre-ART and post-ART periods. Similar to findings from a cohort of married women in Uganda [13], we found that young married women with partners 16 or more years older were at three times greater risk of acquiring HIV infection than those with partners 0–15 years older. However, the current study was not restricted to married women, and controlling for marital status did not affect the association between age-disparate relationships and HIV incidence.

The association between age-disparate relationships and HIV incidence and its contribution to the spread of infection may differ between populations due to differences in the occurrence and effects of underlying and proximate determinants. In Manicaland, greater female education was associated with a reduced frequency of age-disparate relationships. Moreover, concurrent sexual relationships among male partners may mediate the association between age-disparate relationships and HIV incidence, although this could only be evaluated indirectly. Other biological and behavioural factors identified in the proximate determinants framework could also strengthen or attenuate this association, although we did not find evidence for this in the current study possibly due to bias in self-reports of sexual behaviour or insufficient statistical power. The association between age-disparate relationships and HIV incidence could also change over time. This could be due to decreasing HIV incidence in general, decreasing HIV prevalence among male partners, and, particularly, ART scale-up if viral load suppression in older HIV-positive men increases. Again, we did not find evidence for a change in the association between age-disparate relationships and HIV incidence over time in the current study, even in the larger sample of women aged 15–29 years (SDC 6, http://links.lww.com/QAD/B92); although HIV incidence halved over the study period, HIV prevalence among male partners decreased markedly, and ART coverage increased. This may be because ART coverage among the older HIV-positive men who form sexual relationships with young women remained low as ART scale-up was hampered in Zimbabwe by adverse economic conditions and because men tend to lag behind in terms of ART uptake [26]. Therefore, the protective effect of male ART uptake for young women with older partners could increase in the future if growing numbers of infected men initiate ART in accordance with new WHO recommendations on universal ART eligibility.

Differences in these factors may explain the contrast between our results and those from a similar longitudinal study in KwaZulu-Natal, South Africa [14], which did not find an association between age disparity and HIV incidence despite earlier findings of associations with HIV prevalence in South Africa [9] and a recent phylogenetic study that demonstrated that HIV infections in young women can be traced to older men [27]. In Zimbabwe, the scale-up of ART was only intensified recently, the socio-economic context is different, and marriage is more common and occurs at earlier ages [28,29], possibly providing greater exposure to unprotected sex than in casual relationships. The background risk in terms of HIV incidence and HIV prevalence among male partners was also higher in South Africa compared with Zimbabwe [14], so increased risks of HIV acquisition in age-disparate relationships may be obscured by generally high HIV incidence rates, although we did not find evidence for a change in this risk over time in the current study despite a considerable decline in HIV incidence and male HIV prevalence.

Differences in data collection methods or reporting bias can also contribute to different findings between studies. In the current study, informal confidential voting interview methods were used to reduce social desirability bias [30], but some residual bias is likely to remain. Partner's age may be underreported where HIV control programmes have emphasized the dangers of ‘sugar daddies’; alternatively, men may exaggerate their age to facilitate partnership formation. The evidence is mixed [31,32], but, on balance, age-disparate relationships seem most likely to be underestimated, which would tend to make our findings conservative. Other limitations include the use of time period as a proxy for ART uptake in male partners and use of data on only the most recent sexual partner. This may select for long-term partners and, given the relatively long intersurvey periods, may misclassify women as only having age-homogeneous relationships. However, this underestimation of the occurrence of intergenerational relationships is likely to attenuate the association between age-disparate relationships and HIV incidence, making our findings, again, conservative. Time at risk may also have been overestimated given that constant exposure to the last sexual partner was assumed, although excluding participants who reported not having had sex in the past 12 months did not markedly affect the results (SDC 5, http://links.lww.com/QAD/B92). Lack of statistical power may have prevented identification of further important associations within the proximate determinants framework. However, the fact that the increased risk associated with intergenerational relationships in the larger sample of women aged 15–29 years was similar to the risk in the sample of women aged 15–24 years but with less uncertainty suggests that the effect found among women aged 15–24 years was not due to chance.

The results from this study provide evidence that age-disparate relationships can be an important contributor to the continued high HIV incidence rates in adolescent girls and young women in sub-Saharan African populations. Marriage patterns in Zimbabwe are comparable with those in many other parts of sub-Saharan Africa [29], so this result may be more generalizable than those from the South African cohort study. Reducing the prevalence of age-disparate relationships could help to decrease infection rates in this age group [4], and we have found that higher female education was associated with lower occurrence of age-disparate relationships, so structural interventions may be effective in reducing age-disparate relationships. However, age-disparate relationships and associated behaviours occur for deep-rooted cultural and economic reasons, so targeting interventions to reduce the factors that mediate between age-disparate relationships and HIV transmission could be more effective. We found partner concurrency to be a possible mediating factor between age-disparate relationships and HIV infection, and the proximate determinants model proposed in this study could be used to guide future research into such mediating pathways. Interventions addressing these mediating factors could include consistent use of condoms and pre-exposure prophylaxis, and, among male partners, voluntary medical male circumcision, behaviour change programmes (to address partner concurrency), and ART uptake and adherence (to reduce HIV prevalence and infectiousness among male partners). At the same time, programmes that increase female education, economic independence, and risk perception and that engage men to change sex norms will be needed to support these interventions [33].

## Acknowledgements

R.S., S.G., O.M., R.M., and C.N. designed the study. R.S. conducted the literature review. R.S. analysed the data with input from J.W.E., R.R., A.T., and S.G. All authors contributed to interpretation of results and read and approved the final article.

We would like to express our gratitude to everyone who has been involved with the Manicaland Project over the past 20 years. Without those involved in the planning, data collection and processing, and without the study participants, this study would not exist. We would also thank Dr Nadine Schur who developed the methodology for calculating the household wealth index used in this study.

The study was funded by a Wellcome Trust programme grant (084401/Z/07/B).

Data produced by the Manicaland Project can be obtained from the project website: http://www.manicalandhivproject.org/data.html. Here, we provide a core dataset that contains a sample of socio-demographic, sexual behaviour, and HIV testing variables from all six rounds of the main survey. If further data are required, a data request form must be completed (available to download from our website) and submitted to simon.gregson@imperial.ac.uk.

### Conflicts of interest

S.G. declares shareholding in pharmaceutical companies (GSK and Astra Zeneca). For the remaining authors, no conflicts of interests were declared.
